# Host Immunological Factors Enhancing Mortality of Young Adults during the 1918 Influenza Pandemic

**DOI:** 10.3389/fimmu.2015.00419

**Published:** 2015-08-19

**Authors:** Julie L. McAuley, Katherine Kedzierska, Lorena E. Brown, G. Dennis Shanks

**Affiliations:** ^1^Department of Microbiology and Immunology, The University of Melbourne at the Peter Doherty Institute for Infection and Immunity, Melbourne, VIC, Australia; ^2^Australian Army Malaria Institute, Enoggera, QLD, Australia; ^3^School of Population Health, University of Queensland, Brisbane, QLD, Australia

**Keywords:** influenza, pandemic, 1918, pathogenesis, mortality

## Abstract

During the 1918 influenza pandemic, healthy young adults unusually succumbed to infection and were considered more vulnerable than young children and the elderly. The pathogenesis of this pandemic in the young adult population remains poorly understood. As this population is normally the least likely to die during seasonal influenza outbreaks, thought to be due to their appropriate pre-existing and robust immune responses protecting them from infection, we sought to review existing literature for immunological reasons for excessive mortality during the 1918 pandemic. We propose the novelty of the H1N1 pandemic virus to an H1N1 naïve immune system, the virulence of this virus, and dysfunctional host inflammatory and immunological responses, shaped by past influenza infections could have each contributed to their overall susceptibility. Additionally, in the young adult population, pre-exposure to past influenza infection of different subtypes, such as a H3N8 virus, during their infancy in 1889–1892, may have shaped immunological responses and enhanced vulnerability via humoral immunity effects with cross-reactive or non-neutralizing antibodies; excessive and/or ineffective cellular immunity from memory T lymphocytes; and innate dysfunctional inflammation. Multiple mechanisms likely contributed to the increased young adult mortality in 1918 and are the focus of this review.

## Introduction

The 1918–1919 influenza pandemic caused an estimated 50 million deaths (1). In three distinct waves, the pandemic infected a third of the world’s population, with the majority of the deaths occurring during the second wave in late 1918 (2, 3). Disease was characterized by unique, and to date poorly understood, epidemiological and clinical aspects. Victims died either from direct viral infection of the lung (4, 5), or most commonly from secondary bacterial pneumonia (6–8). Unusually, healthy young adults were more likely to die than young children and the elderly, two populations normally most vulnerable during influenza A virus (IAV) outbreaks (9). Fatal cases in the 1918 pandemic peaked in the 1889–92 birth cohorts, corresponding to approximately 28-year-olds (2, 10, 11), a pattern that was observed across the world (9, 12). The extraordinary mortality of young adults during the 1918 influenza pandemic is not currently understood.

Similar to the 1918 pandemic, the 2009 IAV pandemic caused more severe and fatal cases in 30- to 50-year-olds, which constituted up to one-third of patients in hospitals (13–15). Young adults had two to four times the risk of severe outcomes from infection with this virus (H1N1pdm09) than those infected with circulating seasonal influenza (16). The majority of H1N1pdm09 infections caused a self-limited disease and the pandemic was considered the mildest on record. Until we understand the causes of enhanced illness of the young adult population during the 1918 and 2009 IAV pandemics, we are unlikely to be able to realistically estimate the impact of future pandemics. While no single explanation will be relevant to every mortality event in the young adults, we need to understand how their innate and acquired immune status may have combined with viral virulence to enhance mortality.

## Excessive Innate Host Responses Contribute to Influenza Immunopathology

Initial leukocyte infiltration into the lung parenchyma is essential for resolution of virus infection, yet dysregulation of the infiltrating effector cells be a major factor in disease (17, 18). A hallmark of highly pathogenic influenza infections is the ability for the virus to dysregulate innate inflammatory responses, leading to excessive recruitment of effector cells into the lung parenchyma causing severe pulmonary injury and diffuse alveolar damage (19, 20). To elicit cellular infiltrate into the infection site, host pattern recognition receptors (PRRs) must first recognize “danger” signals direct toward the invading IAV, causing release of pyogenic cytokines and chemokines. Excessive or dysregulated secretion can lead to a “storm” of events linked with high-mortality rates (19, 20). Young adults, with robust immune systems, may have been unusually vulnerable to the 1918 IAV due over-exuberant inflammatory responses to infection. As the elderly have less potent inflammatory responses to influenza infection compared to young adults, they may have been somewhat spared from excessive reactions and thus were less likely to succumb to infection.

The recovery of genetic fragments of the 1918 H1N1 pandemic virus and subsequent reverse engineering has enabled a complete reconstruction of the original virus (21). The 1918 pandemic virus was highly pathogenic as infection of monkeys and mice with the reconstructed 1918 H1N1 IAV resulted in acute respiratory distress and death with a pathology that matched lung tissues from victims in 1918 (21–23). Similar features occur with highly pathogenic avian H5N1 and H7N9 IAV infections (20, 24).

Virus infection followed by an extensive influx of macrophages and neutrophils can release large quantities of reactive oxygen species (ROS) contributing to the pathogenesis of lung disease. Mice infected with IAV expressing the virulence protein PB1-F2 matching that of the 1918 pandemic strain had enhanced pulmonary ROS (25), increased cellular infiltrate in alveolar spaces, and were more likely to die from secondary bacterial infections (26) compared to those infected with viruses expressing PB1-F2 proteins from seasonal IAV strains. The type-1 interferons (IFN-α and IFN-β) are the major cytokines that limit influenza replication, with TNFα, IL-1β, and IL-6 recruiting immune cells to the sites of infection and producing inflammation. Studies using mice genetically deficient in inflammatory modulators including tumor necrosis factor receptor (TNFR) and nitric oxide synthase (NOS2) exhibited reduced morbidity and mortality as well as diminished cytokine production in lung tissue following H5N1 and 1918-virus challenge compared to infected wild-type mice (27, 28). The type-1 interferons act on INF-α/β receptors to activate the antiviral signaling cascade, resulting in the production of antiviral proteins, such as MxA (Mx1 in mice). Mice genetically deficient in Mx1, interleukin-1 receptor (IL-1R), or IFNα receptor (IFNAR) exhibited increased viral load and pulmonary inflammation compared to wild-type mice (28–30). The molecular signatures of mice surviving 1918-virus infection reveal that the action of interferon via upregulation of genes involved with apoptosis, ROS production, and cell migration, together with downregulation of genes encoding cytokine and chemokine production associated with viral pathology, such as IL-6 and TNF, is critical to survival (29). As such, type-I IFNs contribute to both resolution of viral load and suppression of immunopathology caused by IAV infections. Inflammatory responses in animal infection models otherwise immunologically naïve toward IAV show that enhancement of inflammation in young adults could have been a major contributor to mortality during the 1918 influenza pandemic.

## Humoral Immunity Enhancing Susceptibility of the Young Adult Population in 1918

**Figure 1:**
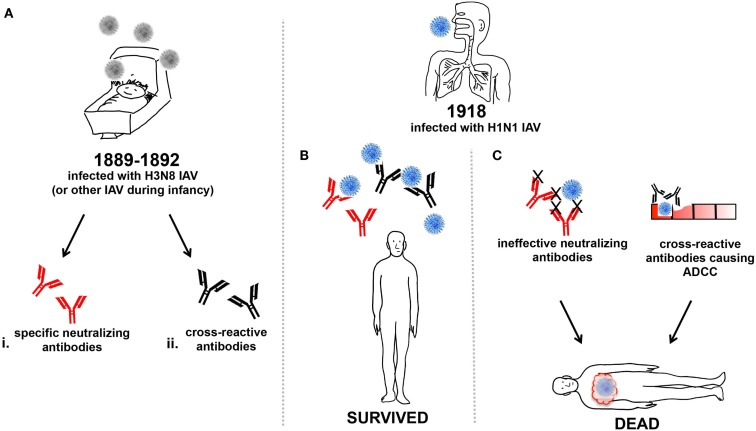
**Humoral influence on vulnerability of young adults to 1918 IAV**. **(A)** Around 1889–92 infants were infected with the H3N8 IAV or other circulating IAV (grey) and generated either (i) neutralizing antibodies (red), or (ii) cross-reactive antibodies (black), or both. The infants from **(A)** that were young adults during the 1918 pandemic and were subsequently infected with H1N1 IAV (blue) may have produced **(B)** specific-neutralizing and/or cross-reactive antibodies enabling effective viral clearance and survival from infection. Or, **(C)** specific-neutralizing antibodies that were ineffective against the heterologous H1N1 IAV strain, and virus was unable to be cleared, resulting in death. Alternatively, the production of cross-reactive antibodies may have also caused ADCC, resulting in cellular damage and inflammatory illness, ultimately contributing to mortality.

Influenza A virus infections during childhood typically induce B-cell memory responses that can adapt to produce antibody protecting against future infection by divergent drift strains of IAVs (31) (Figure 1). Such virus neutralizing antibody responses are typically directed toward epitopes on the globular head of the virus surface glycoprotein hemagglutinin (HA) and can be long-lived. This longevity was particularly evidenced by protection of the elderly against H1N1pdm09 infection, which was attributed to antibodies raised during pre-1960s exposure to a virus of the pandemic lineage (31). The elderly may have survived better than young adults during the 1918 pandemic as they may have been previously exposed to other H1 IAVs (32).

In the absence of specific-neutralizing antibodies, other antibodies that are normally immuno-subdominant can be induced and may be cross-reactive against different IAV subtypes. One such target of subdominant cross-reactive antibody is the viral ion channel protein, M2. The M2 protein is expressed on the virion surface but does not protrude to the level of other glycoproteins, making it a poor viral neutralization target. M2 is more accessible on the surface of infected cells and is thought to enable direct killing of infected cells by antibody-dependent cellular cytotoxicity (ADCC) mechanisms (33–35). Whether anti-M2 antibodies were important during the 1918 pandemic is unknown. Another target of subdominant cross-reactive antibodies are those directed toward the HA stalk (36). Antibodies to the HA-stalk employ various mechanisms of direct and indirect neutralization. By binding to the stalk domain of the HA, the antibody inhibits conformational changes of the HA in the endosome and prevents entry of IAV genomic material into the cytosol, as fusion of the endosomal and viral membranes cannot occur (37). Similar to the cross-reactive anti-M2 antibody, HA-stalk antibodies can induce ADCC (38) and complement-mediated cytotoxicity (39).

In many animal studies, it has been shown that anti-M2 and HA-stalk antibodies induced by vaccination or passive transfer result in viral clearance and protection (35, 38, 40–45). In macaques, weakly immunogenic vaccines did not lead to robust ADCC responses and as such did not contribute to vaccination efficacy (46). Human studies have now revealed cross-reactive HA-stalk antibodies that are broadly neutralizing against divergent IAV strains (e.g., H1N1, H3N2, H5N1, and H7N9) and may protect from infection (41, 45, 47). However, it is important to note that not all individuals are capable of producing HA-stalk antibodies (48). Plasmablasts capable of secreting HA-stalk-specific antibodies have been isolated from healthy adults after H1N1pdm09 vaccination. These cells were produced from already existing memory B cells, which were presumably primed by previous IAV infections (41, 49), a scenario recapitulated by mice in sequential infections (50).

During the 1918 IAV pandemic, prior exposure to previously circulating influenzas would have shaped the memory B cell population to produce a landscape of both direct and cross-protective antibody responses (51) that may have resulted in protection from infection (Figure 1B). Young adults devoid of sufficient memory B cells capable of producing direct and cross-reactive antibodies, due to either their inability to mount such responses or from lack of prior IAV infections, may have fared much worse in 1918 (Figure 1C).

The above assumes that cross-reactive antibodies to HA-stalk or M2 would be beneficial, but evidence also exists that such antibodies may enhance disease. Enhanced respiratory disease can occur when individuals are challenged with a heterologous virus while producing cross-reactive antibodies (52). Pigs vaccinated with an inactivated swine influenza virus showed enhanced pneumonia upon challenge with H1N1pdm09. The vaccine was shown to induce high-titer cross-reactive antibodies against the more conserved HA2 stalk domain but no neutralizing antibodies to the globular head of the HA (53). The pathology associated with non-neutralizing antibodies cross-reacting with heterologous virus was characterized by severe bronchointerstitial pneumonia with necrotizing bronchiolitis and peribronchiolar lymphocytic cuffing (54), which may have resulted from excessive ADCC (Figure 1C).

The phenomenon of vaccine-associated enhanced respiratory disease is reminiscent of that seen in children vaccinated with inactivated RSV or measles virus following exposure to a heterotypic virus, who subsequently suffered enhanced respiratory disease or atypical measles with severe disease (55–57). Reasons for dire outcome include the quality of the antibody elicited toward the virus, the presence of large amounts of non-neutralizing antibody at the time of viral replication, and antibody-mediated activation of the classic complement cascade (56). The young adult population of the 1918 pandemic may have had prior exposure to a double-heterogenic H3N8 IAV during their childhood (32), and may have developed a cross-reactive humoral immunity. It is possible that in some young adults, the cross-reactive antibody responses produced after infection with the 1918 virus actually enhanced subsequent pulmonary disease, for reasons similar to those observed for RSV and measles (55–57). This may be why, compared to children, the young adults were more vulnerable to the 1918 IAV infection.

Whether infection during the initial wave of the pandemic in early 1918 protected one from illness in subsequent waves of the pandemic during late 1918–1919 is not clear despite extensive study (12, 58–61). Young adults infected with the pandemic virus in early 1918 may have had a recall of the memory B cell population boosting the production of both direct and cross-protective antibody responses (51). Subsequent infection during the second or third wave of the pandemic may have resulted in further cross-reactive responses that may have induced ADCC and/or inflammatory disease. In Australian soldiers who could be followed individually, infection in early 1918 appeared to protect against death, but not illness during the subsequent wave occurring later during the 1918 pandemic (12). Additionally, recent Canadian studies showing that seasonal influenza vaccine apparently enhanced illness rates during the 2009 pandemic (62).

A recent study (32) proposes that individuals born earlier than ∼1890–1900 would have had neutralizing antibodies against the 1918 pandemic virus, induced by an emerging H1N1 virus in 1830, or an H1N8 virus in 1847 (32). Those born at the time of the 1889–92 H3N8 pandemic, or shortly thereafter, would not have such neutralizing antibodies and would be highly susceptible to 1918-virus infection. It is further postulated that an H1N8 virus re-emerged in 1900 and may have allowed the children in 1918 some degree of protection. If this did indeed occur, it would account for the troughs in the mortality curves in the young (5–15 years) and older (50–80 years) populations during the 1918 pandemic (32). Similarly, during the 2009 pandemic, about one-third of people born before 1950 had some immunity to the H1N1pdm09 virus, perhaps due to childhood exposure to an antigenically similar IAV (62, 63).

## Cross-Reactive CD8+ T Cell Immunity: Implications for Disease

Pre-existing memory CD8+ T cells established via previous IAV infections can cross-react with common epitopes presented by class I human leukocyte antigen (HLA) complexes on antigen presenting cells and promote rapid viral clearance. Animal (64, 65) and human studies (64, 66–68) have shown that CD8+ T cell-mediated immunity can be directed against highly conserved antigens among different IAV subtypes. More recently, non-conserved peptide epitopes that vary at residues other than those that anchor the peptide within the binding cleft of the HLA can still induce cross-reactive T cells (69). Memory CD8+ T cells can ameliorate infection by heterologous IAVs; however, substantial mutation in IAV peptide epitopes may lead to ineffective recruitment of cytotoxic CD8+ T cells crucial for viral clearance. Alternatively, a lack of capacity to mount any CD8+ T cell response could be equally problematic. The recruitment of cross-reactive CD8+ T cells against IAV varies across different ethnicities and has shown to be dependent upon the capability of expressing the HLA class I alleles that present conserved IAV peptides to elicit cross-protective CD8+ T cells (64).

**Figure 2:**
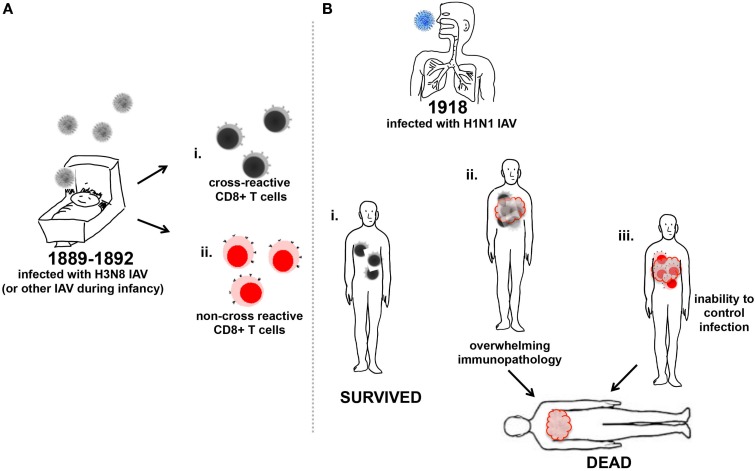
**Memory CD8***+*** T cell influence on increased mortality of young adults to 1918 IAV**. **(A)** Infants were infected with the H3N8 IAV (gray) and generated either memory CD8+ T cells reactive toward (i) antigenically conserved regions of IAV (black/gray cells), or (ii) non-conserved antigenic regions of IAV (red/pink cells). **(B)** Young adults previously infected with IAV in their infancy and produced CD8+ T cells to conserved antigenic regions of the 1918 H1N1 IAV (blue) (i) survived infection as the CD8+ T cells aided viral clearance, or (ii) suffered illness due to the triggering of excessive inflammatory cellular responses to infection and recruitment of an overwhelming number of cross-reactive CD8+ T cells, which may have contributed to death. (iii) Young adults previously infected with H3N8 IAV and produced non-cross-reactive CD8+ T cells in response to heterologous 1918 H1N1 IAV (blue) were unable to control infection and may have become moribund.

Young adults who had survived infection by an IAV in 1890 should have had robust priming of memory CD8+ T cells that conferred some protection from lethal disease during the 1918 pandemic, provided these cells were periodically boosted by intra-pandemic IAV infection (32). Upon infection with the 1918 H1N1 IAV cross-reactive T cell responses would have been rapidly recalled in these individuals and may have protected against their death (70) (Figure 2). Recent studies have shown cross-reactive CD8+ T cell memory pools, generated by previous infection (s) with IAVs could provide some protection against H7N9 IAV infection (64). During 2009, the elderly population had a low infection rate compared to children and young adults, which was thought to be due to T cell immunity and neutralizing antibodies against the extremely conserved immune-dominant epitopes on viral proteins in the 2009 and 1918 H1N1 pandemic strains. Partial cross-reactivity with seasonal H1N1 IAVs that circulated in the 1930s when the elderly population would have been children may have also contributed (69, 71).

Caveats exist for the protective role described for cross-reactive CD8+ T cells. Cross-reactive CD8+ T cells cannot protect the host from initial infection; their target is an infected cell and they must be recruited to the site of infection after recall stimulation. If the heterologous IAV infecting the host presents a strong stimulus via PRRs that trigger excessive inflammatory responses and recruits an overwhelming number of cross-reactive CD8+ T cells, the resulting immunopathology may overwhelm any beneficial effects (10, 72) (Figure 2Bii). In addition, dysfunctional priming of CD8+ T cells may explain why the second wave of the 1918 pandemic appeared more virulent than the first. It has been postulated that the second wave of the 1918 pandemic was caused by a virus that had evolved toward a more pathogenic phenotype than the initially emerging H1N1 IAV (73). However, CD8+ T cells reactive for the immunodominant IAV nucleoprotein (NP) and matrix-1 (M1) produced during first wave of the 1918 pandemic and subsequently recalled upon infection during the second wave in late 1918 may have contributed to an over-exuberant inflammatory response enhancing disease severity (17, 58, 64, 74). As the ability to mount CD8+ T cell responses is linked to highly polymorphic HLA expression, healthy young adults infected in 1918 may have induced highly variable responses that could have been to their detriment due to recruitment of a plethora of non-cross-reactive CD8+ T cells (Figure 2Biii). Additionally, the robustness of the immune system in the young adult population as well as their pre-existing memory CD8+ T cell repertoire may have contributed to the vulnerability of this population over children, who may mounted a smaller repertoire of more specific CD8+ T cells toward the pandemic virus. Given a larger number of previous IAV exposures, the elderly may have mounted a more diverse cross-reactive CD8+ T cell response, but may have achieved clearance of infection without excessive cellular recruitment due to a decreased ability to recruit cells compared to the young adult population. Compared to young adults, the infected elderly CD8+ T cell response to the 1918 H1N1 virus may not have enhanced the pathophysiology of the disease and as such, may have been more effective toward clearing the viral infection.

## Concluding Remarks

The causes of extreme mortality in the young adult population during the 1918 pandemic are still uncertain. Childhood exposure to heterotypic IAV may have shaped humoral and adaptive immunological responses that contributed to the young adult population’s enhanced disease outcomes. Ethnicity resulting in lack of appropriate immunological responses to conserved antigenic sites in the 1918 pandemic IAV may have also contributed to the mortality. PRRs may have induced over-exuberant inflammatory responses enhancing lung pathology and disease. Such mechanisms may collectively explain the increased mortality of young adults during the 1918 influenza pandemic. The enhanced illness in H1N1pdm2009 H1N1-infected young adults demonstrates that we still do not completely understand factors that enhance human vulnerability. We must continue to explore transmission models, virulence factors, and host responses to infection to better understand the pathophysiology of influenza if we are to diminish the impact of any new, highly pathogenic pandemic virus.

## Author Note

The opinions expressed are those of the authors and do not necessarily reflect those of the Australian Defence Force.

## Conflict of Interest Statement

The authors declare that the research was conducted in the absence of any commercial or financial relationships that could be construed as a potential conflict of interest.

## Funding

JM is funded by the National Health and Medical Research Council of Australia (NHMRC) Project Grant 1026619; KK is a CDF2 NHMRC Fellow; KK and LB receive funding from NHMRC Program Grant 1071916; and GS is funded by the Australian Defence Force.
